# *Brucella* Immune Escape: TLR Subversion, Antigen Presentation Destruction and T Cell Disorder

**DOI:** 10.3390/cells14221809

**Published:** 2025-11-18

**Authors:** Hanwei Jiao, Gengxu Zhou, Shengping Wu, Chi Meng, Lingjie Wang, Cailiang Fan, Jixiang Li, Yuefeng Chu

**Affiliations:** 1 The College of Veterinary Medicine, Southwest University, Chongqing 402460, China; zgx973589243@email.swu.edu.cn (G.Z.); chemie@email.swu.edu.cn (S.W.); mengchi@email.swu.edu.cn (C.M.); guolicheng666@email.swu.edu.cn (L.W.); 13308380005@163.com (C.F.); swu_lucky@163.com (J.L.); 2 Animal Epidemic Prevention and Control Center of Rongchang, Chongqing 402460, China; 3 State Key Laboratory for Animal Disease Control and Prevention, College of Veterinary Medicine, Lanzhou University, Lanzhou Veterinary Research Institute, Chinese Academy of Agricultural Sciences, Lanzhou 730000, China

**Keywords:** *Brucella*, immune escape, TLR subversion, antigen presentation destruction, T cell interference

## Abstract

Brucellosis is a severe zoonotic disease caused by *Brucella* infection, which remains prevalent in several regions worldwide and poses a significant public health challenge. The host deploys complex immune mechanisms to combat the pathogen, including the recognition of pathogenic signals, secretion of inflammatory factors, and activation of innate and adaptive immune responses. *Brucella*, as a facultative intracellular pathogen, replicates within host cells and establishes chronic infections through diverse immune evasion strategies. These include subversion of Toll-like receptor (TLR) signaling, disruption of antigen presentation, and interference with T cell responses. This review focuses on *Brucella* species with significant human infectivity, such as *B. melitensis* and *B. abortus*, summarizing their interactions with the host immune system. Recent studies have highlighted TLR pathway inhibition, antigen presentation impairment, and T cell dysregulation as key mechanisms of immune evasion. Understanding these processes is crucial for elucidating *Brucella* pathogenesis and developing novel therapeutic and vaccine strategies against brucellosis.

## 1. Introduction

Brucellosis is a bacterial zoonotic disease caused by *Brucella* infection affecting humans and animals [1]. Brucellosis mainly affects the reproductive tract and can cause abortion and reduced fertility in animals, including endometritis, orchitis, and epididymitis [2]. Humans can become infected by direct contact with infected animals, consumption of contaminated animal products, or exposure to infectious aerosols. Although brucellosis has a low mortality rate, if left untreated, it can develop into a persistent disease with serious complications [3,4,5]. Despite being declared eradicated in some areas, brucellosis remains highly prevalent in some regions worldwide. For instance, according to World Health Organization estimates and studies between 2010 to the present, the global annual incidence is approximately 500,000 new cases, with high burden areas including the Mediterranean basin, the Middle East, West Asia, parts of Latin America, and Africa [4,6,7,8]. The decline in livestock productivity caused by brucellosis results in significant economic losses and affects the development of animal husbandry and international trade [4]. These significant public health and economic burdens underscore the necessity to understand the molecular mechanisms of *Brucella* pathogenesis. A critical aspect of this pathogenesis is immune evasion, which allows the bacterium to establish chronic infections. As a facultative intracellular parasite, *Brucella* lacks capsules and endospores [1]. *Brucella* bacteria colonize mammals, including sheep, cattle, pigs, reptiles, and fish. The main species causing human disease are *B. abortus*, mainly infecting cattle; *B. melitensis*, mainly infecting sheep and goats; *B. suis*, mainly infecting pigs; and *B. canis*, mainly infecting dogs [9].

The immune system is an effective mechanism for the body to respond to pathogens. The body recognizes and eliminates pathogens through various immune mechanisms, while pathogens have evolved multiple strategies to evade the host’s immune system. *Brucella*, which invades host cells and causes long-term chronic infections, can survive and replicate successfully in various cells, forming the basis of its pathogenicity [10]. To counteract the host’s immune system, *Brucella* employs various approaches to evade both innate and adaptive immunity [11,12].

The host’s immune system recognizes various pathogens in tissue through Pattern recognition receptors (PRRs). PRRs detect pathogen-associated molecular patterns (PAMPs) and activate intracellular signaling pathways to induce protective immune responses [13]. The unique structure of *Brucella* PAMPs avoids continuous recognition by the immune system and subsequent strong inflammatory response [9]. CD4^+^ T cells and major histocompatibility complex (MHC) Class II molecules play a key role in host resistance to *Brucella* infection. After *Brucella* infection, the expression of MHC class II molecules decreased in many ways. In addition, *Brucella* can also affect MHC class I molecules. Retaining MHC class I molecules within host cells reduces their expression on the cell surface. These phenomena eventually inhibit the function of effector T cells [14,15].

Studying the interaction between *Brucella* and the host immune system is crucial for the prevention and control of brucellosis. While *Brucella*’s immune evasion mechanisms have been substantially explored, there are still considerable knowledge gaps. Specifically, critical gaps remain in understanding the precise mechanisms of TLR inhibition by effector proteins like BtpA/B, the molecular basis of antigen presentation disruption via MHC-I retention and MHC-II downregulation, and the role of outer membrane proteins in modulating T cell responses. This review aims to address these gaps by consolidating recent advances in Brucella-host interactions to elucidate immune evasion pathways. This article aims to provide a systematic review of the immune evasion mechanisms of *Brucella* and research progress, providing a theoretical basis for the study of *Brucella*’s pathogenic mechanisms.

## 2. The Lifecycle of *Brucella* Within Host Cells

**Figure 1:**
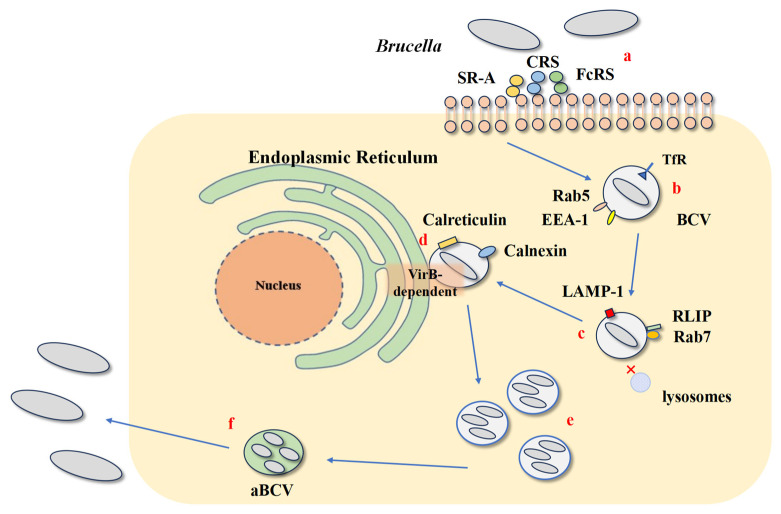
The lifecycle of *Brucella* within host cells. (**a**): *Brucella* enters cells through lipid rafts via complement receptors (CRs), Fc receptors (FcRs), and scavenger receptor A (SR-A), interacts with the cell membrane, and forms *Brucella*-containing vacuoles (BCV). (**b**): BCV interacts with early endosomes and acquires markers GTP-binding protein Rab5, early endosomal antigen 1 (EEA-1), and transferrin receptor (TfR). (**c**): It then interacts with late endosomes and acquires markers GTP-binding protein Rab7, Rab interacting lysosomal protein (RILP), and lysosomal-associated membrane protein1 (Lamp-1). (**d**): BCV avoids fusion with lysosomes (indicated by “X”) and, via the VirB-dependent type IV secretion system (T4SS), fuses with the endoplasmic reticulum (ER) to establish a replication niche. (**e**,**f**): After replication, BCV transforms into an autophagic *Brucella*-containing vacuole (aBCV), which interacts with autophagy-related proteins, promoting bacterial excretion and initiating a new replication cycle.

As a facultative intracellular parasite, *Brucella* can infect both professional phagocytic cells such as macrophages and dendritic cells (DCs), as well as non-professional phagocytic cells such as placental trophoblasts and epithelial cells [16]. Surviving within macrophages and DCs is essential for *Brucella* to evade host immune responses and establish chronic infections [17]. The successful colonization of trophoblasts is central to the reproductive pathology of brucellosis, leading to placentitis and abortion in pregnant animals. While the general intracellular lifecycle (Figure 1) is thought to be conserved, the immune pressures and evasion requirements in a trophoblast, which is not a professional immune cell, may differ significantly from those in a macrophage. The question of whether immune evasion mechanisms are repurposed or specialized in this critical cell type remains an active area of inquiry.

*Brucella* expresses multiple adhesins that regulate its adherence to host cell surface molecules and extracellular matrix components [18]. After attaching to host cells, *Brucella* interacts with complement receptors (CRs), Fc receptors (FcRs), and scavenger receptor A (SR-A) on the host cell surface and enters the host cell via lipid rafts [17,18]. Inside the host cell, *Brucella* interacts with the cell membrane, forming a *Brucella*-containing vacuole (BCV) surrounded by a phagosome [19]. The BCV gains early endosomal markers, such as GTP-binding protein Rab5, early endosomal antigen 1 (EEA-1), and transferrin receptor (TfR), by interacting with early endosomes in the next stage [17]. In the following stage, BCV continues to fuse with late endosomes that have undergone brief acidification, acquiring late endosomal markers, such as GTP-binding protein Rab7, Rab interacting lysosomal protein (RILP), and lysosomal-associated membrane protein 1 (Lamp-1). During this stage, *Brucella* employs multiple factors to prevent BCV from fusing with lysosomes through different mechanisms [17,20]. One lysozyme-like protein, SagA, plays a role in the early stage of *Brucella* intracellular trafficking, thereby avoiding fusion of the BCV with lysosomes. Additionally, the T4SS secretes effector proteins that hijack host GTPases and ER fusion machinery to redirect BCV trafficking away from lysosomes. This reprogramming not only establishes a replicative niche but also directly impairs immune recognition by limiting phagolysosomal maturation and antigen processing, enhancing Brucella’s ability to evade innate immune responses [21]. BCV that escapes lysosomal degradation travels to the ER via the type IV secretion system (T4SS), and fuses with the ER in a COPII-associated small GTPase (Sar1) and GTP-binding protein Rab2-dependent manner. The BCV gains multiple ER markers, such as calnexin, ER calcium-binding protein, and Sec61β, establishing a haven within the ER and replicating, eventually transforming into an autophagic aBCV [4,11,22]. The haven established within the ER allows *Brucella* to evade humoral immunity and keep the bacteria away from macrophage bactericidal activities [11]. Subsequently, aBCV interacts with host autophagy-related proteins, such as Beclin1, ULK1, Atg14, and the IRE1α-UPR signaling axis, enhancing host cell autophagy and promoting the excretion of *Brucella* and the initiation of a new intracellular replication cycle in the infected cell [23,24,25].

## 3. *Brucella* Interferes with the Recognition and Response of the Host’s Innate Immune System

### 3.1. Brucella LPS and Flagella Interfere with TLR Recognition

**Figure 2:**
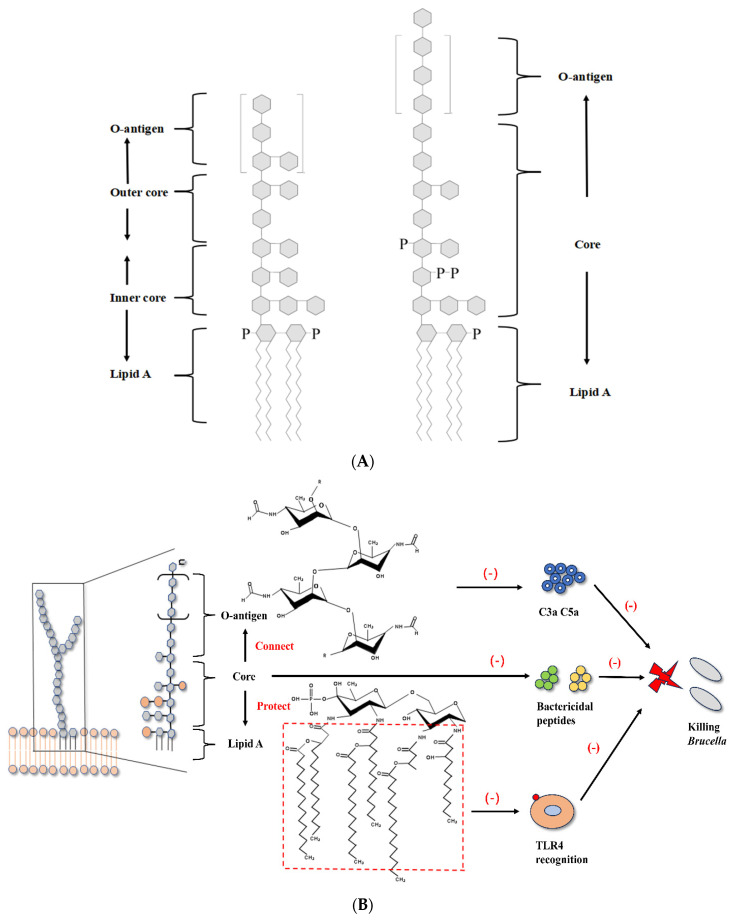
(**A**) Comparison of classical structures of LPS and *Brucella* The structure of *Brucella*. Left one is the structure of a canonical LPS from a typical Gram-negative bacterium, right one is the structure of *Brucella* LPS. (**B**) The lipid A of *Brucella* contains very long-chain fatty acids, which sterically hinder efficient recognition by the TLR4/MD-2 complex, resulting in attenuated pro-inflammatory signaling. The O-PS is predominantly composed of N-formylperosamine residues that lack free hydroxyl groups. This unique structure prevents opsonization by inhibiting ester bond formation with complement C3, thereby blocking the production of C3a and C5a. The core oligosaccharide provides additional steric shielding for lipid A and, due to its positive charge, confers resistance to antimicrobial peptides and complement-mediated killing.

The innate immune system is the first line of defense against pathogens in the body and is crucial for clearing pathogens and controlling infection in the early stages. The innate immune system is related to the adaptive immune system, controlling bacterial replication and creating conditions for the body to produce Th1-type immune responses [11,13]. Different PRR are able to recognize various pathogens in the body, including Toll-like receptors (TLRs), Nod-like receptors (NLRs), Retinoic acid-induced gene I-like receptors (RLRs), and complement [26]. Except for a few wild strains, like most Gram-negative bacteria, the lipopolysaccharide (LPS) of smooth *Brucella* is composed of lipid A, core, and O-specific polysaccharides [9] (Figure 2) (Table 1). *Brucella* LPS and flagella protein interfere with TLR recognition, inhibiting the signaling pathways mediated by TLR4 and TLR5, and reducing the production of pro-inflammatory cytokines [9,27]. The recognition of LPS by the body is usually associated with releasing a series of inflammatory factors, such as TNF-α and IL-12 [28]. TNF-α significantly increases the phagocytic ability of macrophages, and IL-12 induces Th1-type immune responses and produces IFN-γ, which is important for clearing *Brucella* [4].

Lipid A lipopolysaccharide (LPS) is a component of LPS recognized by host TLR4 and is therefore commonly referred to as an “endotoxin.” The class-A LPS of many Gram-negative bacteria induces strong inflammatory responses [29]. However, the lipid A of *Brucella* is a 2,3-di-aminopentadecadiene substituted glucosamine disaccharide with ultra-long acyl chains of C16, C18, C28, and other lengths. This unique structure, particularly the very long-chain fatty acids (VLCFAs), sterically hinders the efficient binding to the TLR4/myeloid differentiation factor-2 (MD-2) complex, making it a weak agonist [4,12,30]. TLR4/MD-2 is considered to be the main receptor complex for recognizing LPS [31]. The non-classical structure of lipid A leads to insufficient recognition of Brucella by the immune system, which inhibits the TLR4-mediated signaling pathway. *Brucella* LPS induces weak inflammatory responses in macrophages and DCs, two cell types that are crucial for the immune system [4]. In the *Brucella* bacA mutant strain that causes abortion, if VLCFAs are absent from lipid A LPS, the mutant strain causes a more severe inflammatory state than the wild-type parental strain. The former has significantly decreased infectivity in BALB/c mice and macrophages [32].

The O-specific polysaccharide of *Brucella abortus* participates in immune evasion by reducing the production of host complement products. The O-specific polysaccharide of bacterial LPS typically contains free hydroxyl groups that facilitate its binding to complement component C3 via ester bonds. In contrast, *Brucella abortus* O-specific polysaccharide is predominantly composed of N-formylperosamine residues, which lack free hydroxyl groups. Due to the absence of this binding target, the O-specific polysaccharide of *Brucella abortus* LPS inhibits the opsonization and the production of pro-inflammatory complement products such as C3a and C5a, avoiding capture by the host immune system [4,12]. The LPS core of *Brucella abortus* also plays a role in immune evasion. Not only does it connect lipid A and O-specific polysaccharide, but its special structure with oligosaccharide side chains also provides steric shielding to lipid A, giving it some degree of protection and inhibiting the binding of the TLR4-MD2 complex with lipid A [33,34]. In addition, this special structure imparts a positive charge to the LPS core, providing some resistance to complement-mediated killing and various antimicrobial peptides [34,35].

The flagella of *Brucella* have also been shown to be involved in the immune evasion of *Brucella*. Host TLR5 recognizes bacterial flagellin, but unlike other bacteria, *Brucella* flagellin lacks amino acid residues that interact with TLR5, so TLR5 does not recognize *Brucella* flagellin and TLR5-mediated inflammatory responses are inhibited [36]. Recent studies using targeted mutagenesis and recombinant protein approaches have further elucidated the immunomodulatory functions of *Brucella* flagellin. For instance, deletion of the fliK gene encoding a key flagellar hook-length control protein in *Brucella* suis S2 significantly enhanced the inflammatory response in infected RAW264.7 macrophages, with upregulated expression of IL-1β, IL-6, IL-18, TNF-α, iNOS, and COX2 [37]. Moreover, recombinant FliK protein was found to inhibit LPS-induced inflammatory responses by downregulating MyD88 and NF-κB expression, decreasing p65 phosphorylation, and suppressing NLRP3 and caspase-1 in the NLRP3 inflammasome pathway [37]. This suggests that *Brucella* flagellin may regulate the host immune system through multiple pathways. While the role of flagella in the initial evasion of TLR5 recognition is clear, further investigation is needed to fully understand how *Brucella* flagellin components like FliK precisely modulate the cytosolic inflammasome response to establish chronic infection.

### 3.2. BtpA and BtpB of Brucella Interfere with TLR Pathways

The activation of TLR requires the participation of multiple adaptor proteins, such as MyD88, TIRAP, TRAM, and TRIF. TLR pathways regulate the secretion of cytokines, the formation of inflammasomes, antigen processing and presentation, and other processes [13]. T4SS plays a role in promoting intracellular replication and establishing chronic infection of *Brucella*. It functions by secreting multiple effector proteins, and T4SS is one of the main virulence factors of *Brucella* [38]. As effectors produced by T4SS, many *Brucella* species express Btp1/TcpB, including *B. melitensis* (TcpB), *B. abortus* (TcpB/Btp1), and *B. ovis* (TcpB) [39]. Btp1 and TcpB are highly homologous, and Btp1/TcpB (also known as BtpA) has a high degree of sequence similarity to the Toll/IL-1 (TIR) domain protein family [5,40,41]. By interfering with the TLR pathway, BtpA inhibits the production of pro-inflammatory cytokines such as IL-12 and TNF-α. In addition, this protein also inhibits the maturation of DCs and the cytotoxic effect of CD8^+^ T cells [42,43].

BtpA is highly homologous to TcpC and TlpA of *E. coli* and enteropathogenic Salmonella, respectively, which have been shown to interfere with TLR signaling pathways [40,44]. TIRAP is a MyD88-dependent adaptor protein for TLR2 and TLR4 signaling, and BtpA was found to mimic the function of TIRAP in TLR signaling by competing with MyD88 and blocking downstream signals. Recombinant BtpA induced ubiquitination and degradation of TIRAP (also known as Mal), interfering with TLR2 and TLR4 signaling [41]. Specifically, the TIR domain dimer of BtpA binds to TIRAP, which may promote TIRAP degradation and inhibit the formation of TIRAP dimers or oligomers. BtpA’s BB loop may mediate the bound TIRAP, can no longer bind to TLR, and induce downstream signal transduction [39]. In addition, complementary experiments with MyD88 fragments showed that besides interacting with TIRAP, BtpA also strongly interacts with MyD88, and the two proteins bind through MyD88’s N-terminal death domain (DD), suggesting that BtpA may target MyD88 to inhibit TLR signaling [45].

**Figure 3:**
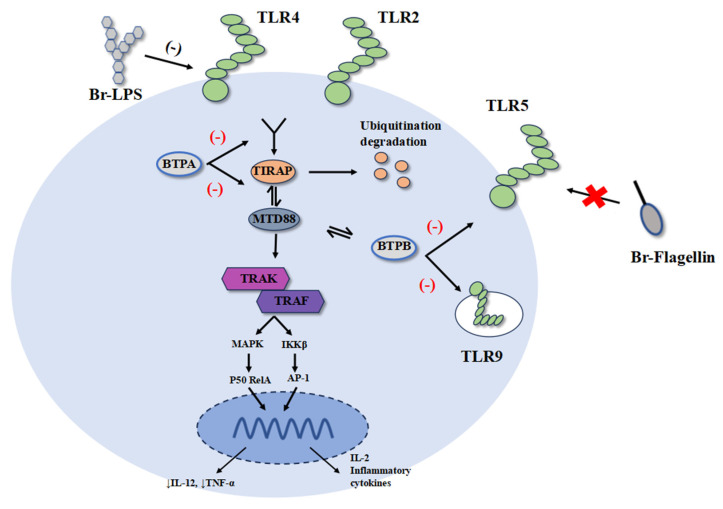
Mechanism of *Brucella* inhibition of TLR signal transduction. LPS attenuates TLR4 recognition, and TLR5 does not recognize flagellin. BtpA induces ubiquitination and degradation of TIRAP in the TLR pathway, blocking MyD88-dependent pathway mediated by TLRs, and reducing the production of pro-inflammatory factors such as IL-12. BtpB is a strong inhibitor of TLR2, TLR4, TLR5, and TLR9 signal transduction, and strongly interacts with MyD88.

BtpB is another TIR domain-containing protein of *Brucella*, which has been demonstrated to be a strong inhibitor of TLR2, TLR4, TLR5, and TLR9 signaling pathways. The specific mechanism of its involvement in *Brucella*’s immune evasion remains to be studied [46,47]. Mutants of BtpB in *Brucella abortus* have been shown to impair TLR signaling, indicating that BtpB negatively regulates pro-inflammatory genes in bovine macrophages [48]. Interestingly, studies have shown that BtpB has a stronger interaction with MyD88 than BtpA, suggesting that BtpB may play a similar role to BtpA in blocking MyD88-dependent TLR signaling pathways (Figure 3). BtpA and BtpB have inhibitory effects on DC maturation and play a significant role in *Brucella*’s immune evasion [47]. Additionally, BtpA and BtpB have been shown to stabilize microtubules, which may impact host cell physiology [49]. Microtubules play a crucial role in intracellular trafficking, including the maturation and transport of vesicles such as the *Brucella*-containing vacuole (BCV). It has been speculated that by stabilizing microtubules, BtpA/B could potentially influence BCV biogenesis and intracellular trafficking, thereby facilitating bacterial replication and immune evasion. Furthermore, microtubules are essential for the transport of MHC molecules to the cell surface during antigen presentation. While direct evidence linking *Brucella*-induced microtubule stabilization to antigen presentation disruption remains limited, it is plausible that this mechanism could impair the delivery of MHC-I and MHC-II molecules to the plasma membrane, contributing to immune evasion. Further studies are needed to elucidate the precise role of BtpA/B-mediated microtubule stabilization in BCV trafficking and antigen presentation.

In recent studies, BtpA and BtpB were found to have NAD^+^ hydrolase activity, which reduces NAD^+^ levels in host cells, suggesting that they not only contribute to *Brucella*’s immune evasion but may also play a role in its pathogenesis [50,51]. Although both effectors exhibit TIR domains and NADase activity, they differ in structure, receptor binding preference, and functional potency. Structurally, BtpA contains a C-terminal tail and a distinct BB loop within its TIR domain, which is critical for TIRAP interaction and degradation [39,41]. In contrast, BtpB lacks this specific C-terminal extension and displays a broader specificity for TLR inhibition, including TLR2, TLR4, TLR5, and TLR9 [46,47]. In terms of receptor interaction, BtpA primarily targets TIRAP and MyD88, disrupting MyD88-dependent TLR signaling [41,45], whereas BtpB shows a stronger binding affinity for MyD88 and may also engage other TIR adaptors, leading to more potent suppression of NF-κB and NLRP3 inflammasome activation in primary macrophages [46,47]. In bovine and goat primary macrophages, BtpB deletion mutants induce significantly higher pro-inflammatory responses compared to BtpA mutants, indicating that BtpB may be a more potent immune modulator in natural host cells [48]. Moreover, both proteins exhibit NAD^+^ hydrolase activity, but their kinetic properties and downstream metabolic impacts may differ, possibly due to variations in their enzymatic domains or host interacting partners such as Protein Disulfide Isomerase A4 (PDIA4), which has been shown to interact with BtpB and influence intracellular NAD/NADH metabolism [51]. Direct side-by-side comparisons in identical cellular systems are still limited, but existing evidence suggests that while both BtpA and BtpB inhibit TLR signaling and possess NADase activity, BtpB may have a broader and more potent inhibitory role, particularly in primary phagocytes. The maintenance of two TIR-domain-containing effectors with overlapping functions likely represents functional specialization rather than mere redundancy. BtpB’s broader TLR inhibition potency and stronger MyD88 binding may equip Brucella to more robustly suppress the initial innate immune response in professional phagocytes. In contrast, BtpA, with its specific targeting of TIRAP, might function in a more cell-type or timing-specific manner. This dual-effector strategy ensures a layered and fail-safe mechanism to subvert TLR signaling across different host niches and infection stages.

### 3.3. Brucella Outer Membrane Proteins Regulate Immunity Through Various Pathways

Outer membrane proteins (Omps) of Gram-negative bacteria play a role in nutrient absorption, cell adhesion, and signal transduction, and in some pathogenic strains, they also participate in evading host defense mechanisms [52]. In addition to maintaining cell membrane integrity, *Brucella*’s outer membrane proteins also play a role in immune evasion. Omp25, Omp16, and Omp19 are the most studied outer membrane proteins for immune evasion. Infection with a *B. suis* Omp25 mutant enhanced macrophage TNF-α production, while the complemented strain can reverse this effect [53]. This phenomenon is similar to that found in *Brucella abortus*, where Omp25 was shown to bind to signaling lymphocyte activation Molecule Family Member 1 (SLAMF1), to inhibit the NF-κB pathway and reduce the production of TNF-α and other pro-inflammatory factors [54]. In addition, in a human macrophage cell line, a pig *Brucella* Omp25 mutant showed an enhanced ability to induce IL-12 production. Recent studies have revealed its molecular mechanism, as Omp25 significantly upregulates miR-155, miR-23b, and miR-21-5p, as well as the programmed cell death receptor Programmed Death-1 (PD-1) [55]. PD-1 is an immune checkpoint receptor expressed on the surface of activated T cells, B cells, natural killer (NK) cells, macrophages, dendritic cells (DCs), and monocytes [56]. Its ligand, PD-L1, is expressed on antigen-presenting cells (APCs), including DCs and macrophages. The binding of PD-1 to PD-L1 transduces an inhibitory signal that downregulates T cell activation and effector functions, a mechanism crucial for maintaining immune homeostasis but exploited in pathological contexts like chronic infection and cancer [56]. In the context of *Brucella* infection, Omp25-mediated upregulation of PD-1 contributes to the inhibition of IL-12 production. This Omp25-mediated upregulation of PD-1 implies that Brucella actively promotes a state of T cell exhaustion, a phenomenon well-characterized in chronic viral infections and cancer, to dampen the adaptive immune response and facilitate persistent infection. By affecting the PD-1 pathway signal transduction, it inhibits IL-12 production at the transcriptional and post-transcriptional levels [55]. Critically, blockade of the PD-1/PD-L1 axis has been demonstrated to restore T cell priming and enhance cytokine production, including IL-12, in various experimental systems [56]. This suggests that the Omp25-PD-1 axis is a significant mechanism by which *Brucella* subverts the adaptive immune response, and its blockade could potentially rescue the impaired IL-12 production and T-cell priming observed during infection.

Brucella Omp16 is a homolog of peptidoglycan-associated lipoprotein (Pal) and highly conserved among Gram-negative bacteria [52,57]. Brucella Omp16 has a certain impact on the production of pro-inflammatory cytokines, as demonstrated in pig brucellosis. Transcriptomics analysis showed that an Omp16-deficient strain significantly upregulated the expression of pro-inflammatory cytokines and chemokines genes including IL-6, IL-11, CCL2, and CCL22, after infecting macrophages [58]. Purified Brucella Omp19 exhibits strong immunomodulatory activity and interacts with TLR2 to influence various host cells [59,60,61]. In addition, Brucella Omp19 possesses protease inhibitor activity and has a protective effect on Omp25, preventing it from being degraded by host proteases [62]. It is important to note that several key immunomodulatory claims regarding Omp19, particularly its TLR2-dependent inhibition of MHC-II expression via IL-6, are based on studies using recombinant protein produced in *E. coli* [14,63]. The acylation state of bacterial lipoproteins is critical for TLR2 recognition, and the acyl moieties transferred by *E. coli* machinery may differ from those of native Brucella Omp19. This could potentially alter its immunostimulatory profile. The extremely low levels of IL-6 observed during wild-type Brucella abortus infection, in contrast to the effects seen with recombinant Omp19, support this notion [5,63]. To conclusively determine the role of native Omp19, an experimental resolution would require the reconstitution of its authentic acylated form, for instance by purifying the lipoprotein from a Brucella background or using an E. coli expression system engineered to mimic Brucella acylation patterns. While S-acylation has been demonstrated to influence the function of other outer membrane proteins, such as Omp25, its direct link to Omp19-mediated immune evasion remains to be firmly established. Furthermore, although recombinant lipoproteins like Omp19 and Omp16 have been explored in subunit vaccine studies, the focus of this review is on the molecular mechanisms of immune evasion; thus, a detailed discussion of Omp acylation and vaccine applications is beyond our present scope. The underlying mechanism of Brucella outer membrane protein immune evasion involves multiple signaling pathways, and the detailed mechanism of their actions remains to be further investigated (Table 2).

## 4. *Brucella* Inhibits Host Antigen Presentation and Adaptive Immune Response

### 4.1. Brucella LPS and Lipoproteins Inhibit MHCII Antigen Presentation

DCs play a significant role in initiating and controlling adaptive immune responses. DCs infected with *Brucella* exhibit low expression of MHCII molecules and co-stimulatory molecules CD80 and CD86, inhibiting DCs maturation and impaired secretion [11]. IFN-γ plays a key role in adaptive immunity and is mainly produced by CD4^+^ T cells. It can activate macrophages and enhance their expression of MHCII molecules, thereby enhancing T-cell antigen presentation. Effector CD4^+^ T cells activated by IFN-γ can increase macrophage bactericidal activity by secreting IFN-γ and assist other T cell subsets such as CD8^+^ T cells to clear the pathogen [64]. A clear mechanism has been found in *B. abortus*, which decreases the expression of IFN-γ-induced MHCII molecules on monocytes and macrophages, inhibiting antigen presentation and interfering with CD4^+^ T cell recognition of infected cells [14,65].

*B. abortus* LPS obstructs the process of antigen presentation by forming macro-structural domains. After infection, *Brucella* is degraded by macrophages, and because the speed of LPS transport within cells is much slower than that of proteins, LPS accumulates continuously in lysosomes. Although the transport of *Brucella* LPS in lysosomes is slow, its resistance to lysosomal degradation and immunological properties remains unchanged [5]. Lysosomes containing *Brucella* LPS circulate to the cell surface, where LPS leaves the lysosome and binds to MHC class II molecules on the surface of macrophages, forming macro-structural domains composed of lipid rafts, LPS, and MHC class II molecules. The formation of these macro-structural domains significantly downregulates the activation of CD4^+^ T cells [64,65,66]. This result may be due to insufficient recognition between cells, and a possible mechanism is that LPS is embedded in the cell membrane through lipids A and binds to MHC II-antigen peptide complexes to form a ternary complex. The unique structure of LPS’s O-specific polysaccharide, composed of N-formyl-peroxyamino residues, may hinder the interaction between MHC class II molecules and antigen peptide complexes, and T cell recognition [5]. However, direct structural evidence demonstrating physical obstruction of the MHC–peptide–TCR interaction by O-PS is still lacking and represents an important area for future research.

Research has shown that *Brucella abortus* lipoproteins, especially Omp19, can reduce the expression of MHC-II molecules and inhibit antigen presentation and processing by interfering with the synthesis pathway of MHC-II [14]. *Brucella abortus* lipoproteins induce the secretion of IL-6 through TLR2, which inhibits IFN-γ-mediated IRF-1 expression. The reduction in IRF-1 expression leads to a decrease in CII class II major histocompatibility complex transactivator(CIITA) transcription, the main transcriptional regulatory factor for MHC-II molecule expression. The reduction in CIITA mRNA leads to a decrease in MHC-II molecule expression and a decrease in CD4^+^ T cell antigen presentation [67,68]. Recombinant Omp19 of *Brucella abortus* compared to heat-killed *Brucella abortus* (HKBA) inhibits the expression of MHC-II molecules and antigen processing to a similar degree, and the inhibition of MHC-II molecule expression depends on TLR2 and is mediated by IL-6 [63].

However, the in vivo relevance of these findings remains uncertain. A major caveat is that the immunomodulatory effects of Omp19 were primarily demonstrated using recombinant protein produced in *E. coli*. The acylation pattern of lipoproteins, which is critical for TLR2 recognition, is dictated by the host bacterial machinery. Therefore, the acyl moieties present on recombinant Omp19 may not faithfully replicate those of the native *Brucella* protein. This is supported by the fact that wild-type *Brucella abortus* infection typically induces extremely low levels of IL-6 [5,64], contrasting with the effects observed with recombinant Omp19. Therefore, the physiological role of native Omp19 in MHC-II inhibition during natural infection requires further validation, necessitating future studies with authentically acylated Omp19 or relevant in vivo models.

### 4.2. Brucella RNA and Btp1/TcpB Inhibit CD8^+^ T Cell Response

CD8^+^ T cells play a vital role in controlling *Brucella* infection. On the one hand, CD8^+^ T cells have cytotoxicity and can eliminate target cells infected with *Brucella*, and on the other hand, the IFN-γ produced by CD8^+^ T cells activates macrophages to kill bacteria [69,70]. Antigen presentation through MHC class I molecules is one way to activate CD8^+^ T cells. MHC class I-deficient mice cannot produce an appropriate CD8^+^ T cell response and control *Brucella* infection slower than wild-type mice [71]. Unlike its effect on MHC class II molecules, *Brucella* infection does not affect the transcription and synthesis of MHC class I molecules. However, it induces MHC class I molecules to accumulate in the Golgi apparatus, which leads to decreased expression of MHC class I molecules on the cell surface and inhibition of antigen presentation [15]. In addition, another mechanism of immune escape has been found in *Brucella melitensis*. The effector protein produced by the T4SS system of *Brucella melitensis* can reduce immune synapses’ effectiveness, ultimately inhibiting the cytotoxic effect of CD8 ^+^ T cells [7].

The RNA and RNA degradation products of *Brucella abortus* cause the retention of MHC class I molecules in the Golgi apparatus, which participates in the inhibition of IFN-γ-induced MHC class I expression in human monocytes and macrophages [66,72]. Intracellular retention of MHC class I molecules is a well-established immune evasion mechanism many viruses have utilized and a similar mechanism has been observed in *Brucella abortus* [73,74,75]. After infection, *Brucella abortus* replicates within macrophages, and RNA degradation products are released into the endosomes containing *Brucella abortus.* The secretion of EGF-like ligands, such as EGF and TGF-α, is induced through the TLR8-mediated signaling pathway. These ligands are secreted outside the cell and bind to ErbB receptors on the cell surface. The ErbB receptors that are bound send signals through the ERK1/2 pathway. This results in the retention of MHC class I molecules. MHC class I molecules stay in the Golgi apparatus of the cell and cannot reach the cell surface, eventually inhibiting antigen presentation [76,77]. While using RNA as a signal risks detection by other RNA-sensing pathways, *Brucella* may mitigate this by confining its RNA within endosomal compartments, thereby specifically engaging TLR8 while limiting exposure to cytosolic sensors and minimizing a broader antiviral immune activation. Crucially, pharmacological inhibition of the EGFR or the ERK1/2 pathway has been demonstrated to normalize the surface expression of MHC-I molecules on *Brucella abortus*-infected macrophages [77,78]. This restoration of antigen presentation is functionally significant, as it sufficiently rescues the ability of antigen-specific CD8^+^ T cells to recognize and kill infected target cells in vitro [77]. This key evidence directly establishes the EGFR/ERK1/2 pathway as a critical mediator of *Brucella*’s strategy to evade cytotoxic T lymphocyte (CTL) killing. Studies have shown that the inhibition of Golgi acidification is associated with the retention of MHC class I molecules [15]. Interestingly, it has been demonstrated that *Brucella abortus* interacts with the Golgi apparatus through its type IV secretion system, regulating vesicular transport and promoting the biogenesis and replication of the *Brucella*-containing vacuole (BCV) [38]. Suggesting that the type IV secretion system of *Brucella abortus* may play a role in the process of MHC class I retention.

**Figure 4:**
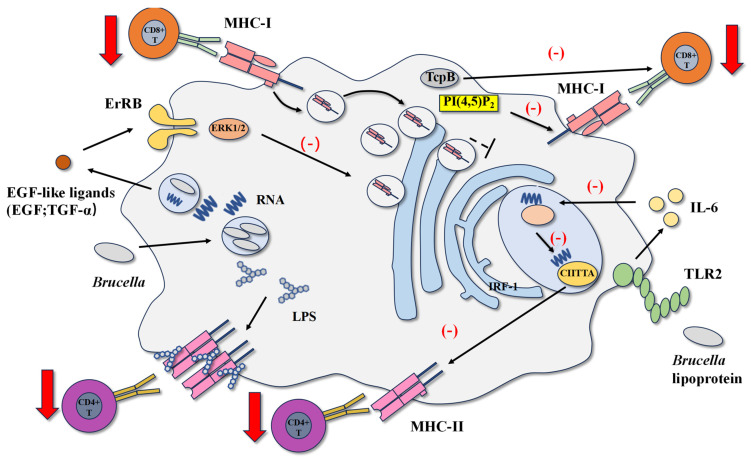
*Brucella* regulates MHC class I and II molecules, impedes T-cell recognition, and interferes with antigen presentation. *Brucella* RNA and its degradation products activate the TLR8-EGFR-ERK1/2 signaling axis, leading to the retention of MHC-I molecules within the Golgi apparatus. This prevents their normal trafficking, inhibiting antigen presentation to CD8^+^ T cells. *Brucella* LPS accumulates in lysosomes, circulates to the cell surface, and forms macrodomains with MHC-II molecules, hindering CD4^+^ T cell recognition. Lipoproteins signal via TLR2 to downregulate CIITA and subsequently MHC-II expression. In antigen-presenting cells (APCs), the effector protein TcpB sequesters phosphatidylinositol 4,5-bisphosphate (PI(4,5)P_2_) away from the immunological synapse, impairing its function. A direct effect of TcpB on CD8^+^ T cells has also been proposed, contributing to the inhibition of cytotoxic T lymphocyte (CTL) activity.

As previously mentioned, Btp1/TcpB of *Brucella* has been shown to interfere with TLR pathways effectively. Recent studies have discovered new mechanisms in inhibiting CD8^+^ T cell function, where the TcpB of *B. melitensis* plays an important role. Similarly to TIRAP, TcpB interacts with inositol phosphates via its N-terminal domain [78]. Phosphatidylinositol 4,5-bisphosphate (PI(4,5)P_2_) is a signaling lipid concentrated in the immunological synapse of antigen-presenting cells. Apart from maintaining membrane integrity, it regulates multiple cellular activities such as phagocytosis, cell movement, and vesicular transport [79]. TcpB contains a transmembrane domain, which allows it to translocate from the vesicular *Brucella* to the cytoplasm of the infected cell, bind to phosphatidylinositol 4,5-bisphosphate, isolate it from the cell membrane, and move it away from the immunological synapse. The decreased immunological synapse effect will inhibit the function of effector CD8^+^ T cells. Additionally, TcpB can directly act on CD8^+^ T cells and inhibit their function [7] (Figure 4).

## 5. Conclusions

The body has developed various immunological mechanisms to recognize and eradicate invading infections. On the other hand, many pathogens have evolved effective ways to evade the immune system. *Brucella* uses a variety of strategies to regulate the host’s immune system for immune escape (Table 3), bypassing the host’s immune system and ultimately developing into a chronic infection. *Brucella* blocks TLR-mediated signaling pathways in various ways, reducing the production of pro-inflammatory cytokines such as IL-12, TNF-α, and IFN-γ, which initiate innate and adaptive immune responses and participate in antigen presentation. *Brucella* inhibits the maturation of DCs, interferes with antigen presentation, blocks T cell recognition, and suppresses T cell activity in multiple ways.

Although some of the immune evasion mechanisms of *Brucella* have been elucidated, many questions are still waiting to be answered about the mechanisms by which *Brucella* “hides” in the body. As a facultative intracellular parasite, *Brucella* is known as the “master of stealth” due to its ability to hide in host cells. Revealing its immune evasion mechanisms will help study its pathogenesis and develop new and effective treatments for brucellosis.

Elucidating the causal relationship between specific immune evasion mechanisms and bacterial persistence requires a combination of well-designed in vitro and in vivo experiments. In vitro, intracellular bacterial counts at various time points provide a direct measure of the bacterial load within host cells. To dissect the role of host factors, gene silencing or knockout in relevant cell lines followed by analyses of cell viability, cytokine production, and signaling pathway activation (via qPCR, Western blot) can pinpoint molecular consequences. For bacterial virulence factors, isogenic mutant strains of *Brucella* are indispensable tools to compare against wild-type strains in their ability to modulate host cell functions and establish replicative niches.

These in vitro findings must be corroborated in vivo to establish physiological relevance. Key in vivo readouts include bacterial organ burden and the assessment of chronic infection establishment in animal models. The causality between a highlighted evasion mechanism and bacterial persistence can be rapidly tested in vivo through two primary, tractable approaches: first, by infecting host-specific gene knockout mice with wild-type *Brucella* and comparing the resulting bacterial load and immune response to those in wild-type mice; and second, by treating wild-type mice infected with wild-type *Brucella* with specific pharmacological inhibitors and evaluating the therapeutic effect on bacterial clearance.

Building on these experimental frameworks, several research directions appear particularly promising for advancing our understanding and developing novel countermeasures. One priority is to investigate the potential of druggable host pathways, such as the EGFR/ERK axis, to restore MHC-I antigen presentation and CD8+ T cell-mediated clearance of *Brucella*. Another compelling direction is the development and application of specific inhibitors targeting the NADase activity of BtpA/B effector proteins to determine the contribution of this metabolic subversion to chronic infection. Finally, a broader exploration to define non-TLR pattern recognition receptors engaged by *Brucella* PAMPs could reveal previously unknown layers of the host immune response and bacterial counter-strategies. The integration of these mechanistic studies holds significant promise for identifying new therapeutic targets and vaccine strategies against brucellosis.

## Figures and Tables

**Table 1:** Summary of Brucella LPS structural features, immune receptors affected, and in vivo outcomes.

LPS Component	Structural Feature	Receptor Affected	In Vivo Outcome
Lipid A	Presence of very long-chain fatty acids (VLCFAs; e.g., C28)	TLR4/MD-2 (weak agonist)	Attenuated pro-inflammatory response; facilitates chronic infection
Core	Positively charged; branched oligosaccharides	TLR4/MD-2 (steric hindrance); Antimicrobial peptides; Complement	Resistance to cationic antimicrobial peptides and complement-mediated killing
O-Polysaccharide (O-PS)	Composed of N-formylperosamine, lacking free hydroxyl groups	Complement component C3	Impairs complement activation and opsonization; reduces phagocyte recruitment

**Table 2:** Summary of Key *Brucella* Effector Proteins and Their Immune Evasion Mechanisms.

Effector Protein	Target/Receptor	Main Action/Effect	Effector Protein
BtpA (TcpB)	TIRAP, MyD88	Degrades TIRAP; inhibits MyD88-dependent TLR2/4 signaling; reduces IL-12 and TNF-α production; inhibits DC maturation and CD8^+^ T cell function	BtpA (TcpB)
BtpB	MyD88, TLR2/4/5/9	Strong inhibitor of multiple TLR pathways; interacts strongly with MyD88; suppresses NF-κB and NLRP3 inflammasome; possesses NAD^+^ hydrolase activity	BtpB
Omp25	SLAMF1, PD-1 pathway	Binds SLAMF1 to inhibit NF-κB; upregulates miR-155, miR-23b, miR-21-5p and PD-1; inhibits IL-12 production and T cell priming	Omp25
Omp16	Macrophage signaling	Deficiency upregulates pro-inflammatory cytokines (e.g., IL-6, CCL2); highly conserved peptidoglycan-associated lipoprotein	Omp16
Omp19	TLR2	Recombinant form inhibits MHC-II via IL-6/IRF-1/CIITA axis; protects Omp25 from proteolysis; may modulate autophagy	Omp19

**Table 3:** Summary of Key Immune Evasion Mechanisms Employed by Brucella.

Mechanism Category	Key Molecule/Component	Main Action/Effect	Selected References
TLR Recognition Subversion	Lipopolysaccharide (LPS)	Weak TLR4/MD-2 agonist due to VLCFAs in Lipid A; O-PS impairs complement opsonization.	[4,9,12,31,33,34]
	Flagellin	Lacks TLR5-interacting residues, evading detection and pro-inflammatory responses.	[36]
	BtpA/TcpB	Mimics TIR domain, degrades TIRAP, interacts with MyD88, inhibiting TLR2/4 signaling.	[39,41,45]
	BtpB	Broad TLR inhibitor (TLR2/4/5/9); strong MyD88 interaction; possesses NADase activity.	[46,47,50,51]
Innate Immune Modulation	Outer Membrane Protein Omp25	Binds SLAMF1, inhibits NF-κB; upregulates miRNAs and PD-1, inhibiting IL-12 production.	[53,54,55]
	Outer Membrane Protein Omp16	Deficiency upregulates pro-inflammatory cytokines in macrophages.	[58]
	Outer Membrane Protein Omp19	TLR2 agonist; recombinant form inhibits MHC-II via IL-6; protects Omp25 from proteolysis.	[8,14,63,64]
Antigen Presentation Disruption	LPS (MHC-II interference)	Forms macrodomains with MHC-II on macrophage surface, hindering CD4^+^ T cell recognition.	[5,65,66,67]
	Lipoproteins (e.g., Omp19)	TLR2/IL-6 dependent downregulation of CIITA and MHC-II expression.	[14,64,67]
	Bacterial RNA (MHC-I interference)	Retains MHC-I molecules in the Golgi via TLR8-EGFR-ERK1/2 pathway.	[15,73,76,77]
T Cell Response Interference	Btp1/TcpB (CD8+ T cells)	Sequesters PI(4,5)P_2_ at immunological synapse; directly impairs CD8^+^ T cell function.	[7,79]
	Omp25 (Indirect T cell inhibition)	Upregulates PD-1 on macrophages, contributing to T cell exhaustion.	[55]

## Data Availability

Data sharing is not applicable to this article as no datasets were generated or analyzed during the current study.
